# Production of foot-and-mouth disease virus SAT2 VP1 protein

**DOI:** 10.1186/s13568-019-0938-7

**Published:** 2020-01-07

**Authors:** Mpho Victoria Mamabolo, Jacques Theron, Francois Maree, Michael Crampton

**Affiliations:** 1 Department of Biochemistry, Genetics and Microbiology, University of Pretoria, Pretoria, South Africa; 2 Agricultural Research Council, Pretoria, South Africa; 3 Council for Scientific and Industrial Research, Pretoria, South Africa

**Keywords:** FMDV, VP1, Recombinant, Expression, Fermentation, Diagnostics

## Abstract

The seven serotypes of foot-and-mouth disease virus (FMDV) differ on the surface exposed regions on the VP1, 2 and 3 proteins. Amongst the three, the VP1 protein has been produced the most for use in serotyping assays for some of the Euro-Asian serotypes. In this study the VP1 protein of the FMDV SAT2/ZIM/7/83 was expressed in *Escherichia coli* BL21 cells in Luria broth and EnPresso^®^ B media in shake flasks. Production was further developed and the VP1 protein was produced at 2.15 g L^−1^ in fed-batch fermentations at 2 L scale. The protein formed insoluble inclusion bodies that were isolated, denatured and refolded. When tested in ELISA, the protein was found to be highly reactive with serum from a SAT2 vaccinated guinea pig, and not reactive to SAT1 and SAT3 antisera. These results open avenues to evaluate recombinantly expressed VP1 proteins for differentiation of the three Southern African Territories serotypes of FMDV that co-occur in Southern and East Africa. In addition, this could mitigate the need for employing virus as reagent, or having to raise reagent antibodies.

## Introduction

Over the past 30 years, there have been efforts to produce the VP1 protein and peptides of the foot-and-mouth disease virus (FMDV) for application in diagnostics or as a vaccine for controlling the economically important and highly infectious foot-and-mouth disease (FMD). The VP1 protein contains most of the virus’ neutralization sites, has serotype-determining regions, and is involved in the binding of the virus to host cells (Cheung et al. 1983; Parry et al. 1990; Jackson et al. 2003). For the past 60 years, the disease has been controlled with an inactivated virus vaccine. Traditionally, diagnosis of FMD involve methods that make use of the inactivated virus antigen or, raising of reagent antibodies in laboratory animals. Both vaccine and diagnostic reagents must be produced in high containment facilities to avoid the risk of virus escaping from production facilities to the surrounding environment (Cottam et al. 2008).

The interest in VP1 emanated from studies that demonstrated that the isolated protein could induce a neutralizing response in animals (Laporte et al. 1973; Wang et al. 2015). Structural and functional studies attributed the immunogenicity of VP1 to the presence of 11 T-cell epitopes and three of five virus neutralizing sites (Crowther et al. 1993; Davidson et al. 1995; Grubman and Baxt 2004; Ko et al. 2010; Grazioli et al. 2006; Mahapatra et al. 2012; Rodriguez et al. 1994; Volpina et al. 1999). The VP1 protein also plays a significant role in serotype specificity in the seven FMDV serotypes (Cheung et al. 1983; Jackson et al. 2003; Parry et al. 1990). This led to the search for inexpensive and alternative methods of diagnosing FMD. The VP1 protein has been recombinantly expressed in various hosts including plants (Li et al. 2006), yeast (Shi et al. 2006), baculovirus (Li et al. 2008), mammalian cells (Moraes et al. 2002) and bacteria (Jung et al. 2013). Successful challenge experiments where animals were immunized with recombinantly expressed VP1 have been reported. The immune response of these experimental animals and natural hosts produced virus neutralizing antibodies and protection from viral challenge (Grubman et al. 1993; Wigdorovitz et al. 1999a, b).

For application in diagnostics, the C-terminal half of the VP1 protein of the FMDV serotype O was expressed in *Escherichia coli*, and antibodies were raised against the protein in guinea pigs. The purified protein and antibodies were then shown to be specific to serotype O when tested against serotypes A, O, C and Asia-1 in an enzyme-linked immunosorbent assay (ELISA) and latex agglutination test (Suryanarayana et al. 1999). The reliable differentiation of serotype-specific antibodies with recombinant VP1 was demonstrated by Wenger et al. (1999) and by Chen et al. (2016) in an indirect ELISA and Luminex assay respectively. Recombinant VP1 protein was also used as antigen on an immunochromatographic strip for specific rapid detection of FMDV serotype O (Yang et al. 2010). Although the VP1-encoding gene has been targeted in a PCR assay aimed at detecting SAT-type viruses (Bastos 1998), the use of the VP1 protein of the three Southern African Territories (SAT) serotypes of FMDV, viz. SAT1, SAT2 and SAT3, in serology has not been evaluated. Reported here is a production method for the VP1 protein of FMDV SAT2/ZIM/7/83 in shake flask under batch and fed-batch mode. The production process was upscaled to 2-L fermenters. Isolated and refolded recombinant VP1 was evaluated for specificity of the produced protein against SAT1 and SAT3.

## Materials and methods

### *E. coli* strains, plasmids and growth conditions

An *E. coli* DH10B [F^−^ mcrA Δ(*mrr*-*hsd*RMS-*mcr*BC) Φ80d*lac*ZΔM15 Δ*lac*X74 *end*A1 *rec*A1 *deo*R Δ(*ara*,*leu*)7697 *ara*D139 *gal*U *gal*K *nup*G *rps*L λ^−^] strain used for subcloning was obtained from Invitrogen. *E. coli* BL21 (DE3) (F^−^*omp*T hsdSB (rB^−^ mB^−^) gal dcm) used for protein production was obtained from Stratagene. The pBluescript SK (pSK), (Amp^R^) and pET28a, (Kan^R^) plasmids were obtained from Stratagene and Novagen, respectively. *E. coli* cultures were grown in Luria Bertani (LB) medium (5 g L^−1^ NaCl, 5 g L^−1^ tryptone, 10 g L^−1^ yeast), pH 7, at 37 °C, 200 rpm with 100 µg mL^−1^ ampicillin and 50 µg mL^−1^ kanamycin. For protein expression in shake flasks, EnPresso^®^ B (Krause et al. 2010; Ukkonen et al. 2013) (Biosilta) and LB media were evaluated. The former was re-constituted by dissolving EnPresso^®^ B medium tablets in sterile water as per the manufacturer’s instructions.

### Serum samples

All anti-FMDV and naïve guinea pig serum samples were obtained from the Agricultural Research Council, Onderstepoort Veterinary Institute. The anti-FMDV serotypes SAT1, 2 and 3 sera were from guinea pigs that were vaccinated with FMDV SAT1/SAR/9/18, SAT2/ZIM/7/83 and SAT3/KNP/10/90, respectively. Naïve serum was from guinea pigs with no history of FMDV infection.

### DNA techniques

Plasmid DNA was isolated with a Zyppy™ Plasmid Miniprep Kit (Zymo Research) according to the manufacturer’s instructions. Restriction enzymes were used as specified by the manufacturers (Epicentre and Thermo Scientific). Plasmid DNA was transformed into *E. coli* by electroporation (Dower et al. 1988). A 615-base pair (bp) gene encoding the VP1 protein of FMDV SAT2/ZIM/7/83 (GenBank Accession no: DQ009726) was codon-optimized (GenBank Accession no: SAMN11897314) for expression in *E. coli* and synthesized by GenScript Corp (http://www.genscript.com) (Additional file 1: Fig. S1). The gene was PCR amplified with KAPA HiFi DNA polymerase (KAPA Biosystems) and VP1F (5′-ACTGGATCCGTTGTTACCACCGACCCGTCT-3′; *Bam*HI site underlined) and VP1R (5′-ACTGCGGCCGCCTGTTTTTCAACACCGATCGG-3′; *Not*I site underlined) primers. Transformants in pSK plasmid (harbouring the amplified gene) were screened by colony PCR with amplification primers (VP1F and VP1R); in a pET28a plasmid vector, they were screened with T7 promoter (5′-TAATACGACTCACTATAG-3′) and VP1R primers. KAPA 2G DNA polymerase (KAPA Biosystems) was used in the screening. The integrity of the cloned *VP1* gene was verified by Sanger DNA sequencing, performed by Inqaba Biotechnical Industries.

### Construction of pVP1

The PCR amplified *VP1* gene was blunt-end cloned into *Eco*RV-linearized pSK plasmid DNA with the Fast-Link™ DNA ligation kit (Epicentre) and transformed into *E. coli* DH10B to generate pSK-VP1. The pSK-VP1 plasmid was then digested with *Bam*HI and *Not*I to recover the *VP1* gene, which was ligated into pET28a digested with the identical restriction enzymes to yield pVP1. The recombinant plasmid was electroporated into *E. coli* BL21 (DE3) to generate the bacterial strain EC-VP1 that was used in all subsequent experiments. The EC-VP1 bacterial strain was stored as 0.5 mL glycerol stocks at − 80 °C until use.

### Production of the VP1 protein in shake flasks

Production of the VP1 protein in shake flasks was evaluated in LB and EnPresso^®^ B (Sigma, USA) media each in triplicate. For both media, the cultures were induced with isopropyl β-d-1-thiogalactopyranoside (IPTG) to a final concentration of 1 mM, following which samples were taken bihourly for 24-h to measure growth at OD_600_ and protein production. For the latter, samples from the three flasks were pooled for analysis of protein expressed at each time point.

### Expression of VP1 in fed-batch fermentation

Fermentation of EC-VP1 was carried out in triplicate in 2-L Infors fermenters containing 1.5 L of medium composed of 2.5 g L^−1^ citric acid, 5 g L^−1^ NH_4_NO_3_, 2 g L^−1^ (NH_4_)_2_SO_4_, 4.5 g L^−1^ Na_2_HPO_4_∙2H_2_O, 14.6 g L^−1^ KH_2_PO_4_, 20 g L^−1^ yeast extract, 2% [w/v] glucose, 1 mL L^−1^ antifoam, 0.05 g L^−1^ kanamycin and 5.23 mL L^−1^ trace element solution (0.4 g L^−1^ CaCl_2_∙2H_2_O, 16.7 g L^−1^ FeCl_3_∙6H_2_O, 0.15 g L^−1^ MnCl_2_∙4H_2_O, 0.18 g L^−1^ ZnSO_4_∙7H_2_O, 0.125 g L^−1^ CuCl_2_∙2H_2_O, 0.18 g L^−1^ CoCl∙6H_2_O and 20.1 g L^−1^ Na_2_EDTA). The temperatures were maintained at 37 °C pre-induction, and reduced to 30 °C post-induction. The pH in the vessels was maintained at 7 by addition of NH_4_OH (30% N) or 2 M H_2_SO_4_. The dissolved oxygen (DO) was maintained at or above 40% saturation by increasing agitation speed in the batch phase. The percentage of dissolved oxygen (DO) was then used as an indirect feedback control during fed-batch process; a decrease of < 40% in DO triggered the release of the glucose or booster feed. The initial charge of glucose in the fermenters was 2.2 g. The glucose was fed when the initial charge was depleted until induction. After induction, the glucose booster was fed throughout the production phase.

Protein expression was induced 7 h post inoculation with a final concentration of 1 mM IPTG. A booster (24 g L^−1^ yeast extract, 17% [w/w] glucose, 12 g L^−1^ meat free tryptone and 1.5 g L^−1^ MgSO_4_) was fed throughout the protein production phase. Samples were taken from the bioreactors at different time points and analyzed for OD_600_ and dry cell weight (DCW), whereas protein yields were analyzed at harvest.

### Protein extractions and analysis

To analyze expression of the VP1 protein in *E. coli* BL21, cells were first lysed. For this, stored cell pellets were resuspended in a Bacterial Protein Extraction Reagent, B-PER™ (Thermo Scientific), as per the manufacturer’s instructions. Pelleted insoluble proteins were solubilized in solubilization buffer (0.1 M Tris, pH 8, 8 M urea) of equal volume to the starting culture volume and 10 µL was analyzed by sodium dodecyl sulfate polyacrylamide gel electrophoresis (SDS-PAGE), as described by Laemmli (1970). The concentration of VP1 was determined by scanning the gel and quantifying the appropriate protein band with BioRad Image lab 4.1 software. Bovine serum albumin (BSA) was included on the gel as a standard. For western blot, a 1 µL sample was loaded onto the polyacrylamide gel for analysis.

### Inclusion body extraction and refolding

To extract inclusion bodies, the cell pellet from a 50-mL culture was suspended in B-PER and centrifuged as described above, unless specified otherwise. The pellet was resuspended in DOC buffer (50 mM Tris, pH 8, 2% [w/v] sodium deoxycholate, 2 M urea, 5 mM EDTA) and then centrifuged. The pellet was then washed in 50 mM Tris, pH 8, and then solubilized in 5 mL of 8 M urea and left overnight on a shaking platform. The sample was centrifuged and the supernatant diluted to the original 50 mL starting culture volume with TNGA buffer (100 mM Tris, pH 8, 100 mM NaCl, 1% glycerol, 1 M l-Arginine), and centrifuged at 18000*g* for 30 min, 4 °C. The supernatant containing refolded protein was loaded on an SDS–polyacrylamide gel for analysis or stored at − 20 °C until use.

### Western blot analysis

To confirm expression of recombinant VP1, western blot analysis was performed under denaturing conditions according to Gallagher et al. (1997). The immobilized protein was probed with anti-6X His tag antibodies conjugated to horseradish peroxidase (HRP) obtained from Sigma-Aldrich.

### Specificity of recombinant VP1

The specificity of the recombinant VP1 was evaluated by direct ELISA following extraction, isolation and refolding as detailed above. For this, half of a Maxisorp Immunoplate (Nunc) was coated overnight at 4 °C with 100 μL of a 100 mg L^−1^ VP1 diluted 1:100 in carbonate/bicarbonate buffer pH 9.6. The other half of the plate was incubated with only coating buffer. All wash steps were repeated three times for a total of four washes with PBS-T buffer (137 mM NaCl, 2.7 mM KCl, 8 mM Na_2_HPO_4_, 1.46 mM KH_2_PO_4_, 0.05% Tween-20). After washing, all the wells were incubated with 5% casein at 37 °C for 2 h to block non-specific binding. Sera was added to the wells in octuplicate. The sera was from guinea pigs that were vaccinated with FMDV SAT1/SAR/9/18, SAT2/ZIM/7/83, SAT3/KNP/10/90 and from naïve guinea pigs respectively, diluted 1:1000; 100 μL each added to the coated and uncoated wells, respectively, and incubated at 37 °C for 2 h with mild agitation. The plate was washed and 50 μL of a goat anti-GP IgG F(ab’)2 HRP conjugated secondary antibody (Merck), diluted 1:80, was added and the plate incubated at 37 °C for 2 h. After washing, the ELISA plates were developed by addition of 100 μL substrate/chromogen solution [30% (w/v) H_2_O_2_/4 mM TMB in substrate buffer (0.1 M citric acid 300 monohydrate, 0.1 M tri-potassium citrate; pH 4.5)] for 10 min. The reaction was stopped with 50 μL 1 M H_2_SO_4_ and the absorbance read at 450 nm using a Labsystems Multiscan Plus photometer. Absorbance readings from the uncoated side were deducted from the coated side to normalize background activity. The results were analyzed by one-way ANOVA.

## Results

### VP1 protein expression

**Fig. 1:**
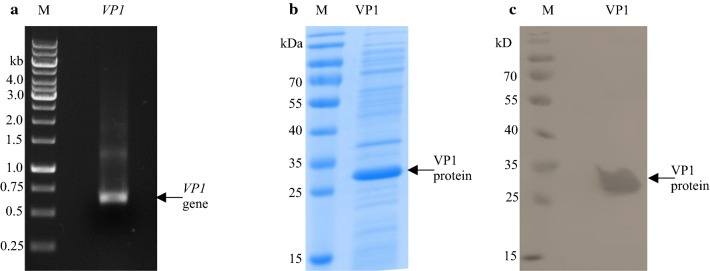
Expression of the *VP1* gene of the FMDV SAT2/ZIM/7/83 in *E. coli* BL21 (DE3) cells. The 615-bp *VP1* gene was PCR amplified and electrophoresed on a 1.5% agarose gel (**a**). The GeneRuler^TM^ 1 kb DNA Ladder (Thermo Scientific) was loaded as a marker (M). The recombinant VP1 protein was produced by EC-VP1 after induction with a final concentration of 1 mM IPTG in EnPresso B medium (**b**). After cell lysis with BPER, the insoluble fraction was analyzed on a 15% SDS–polyacrylamide gel, the expressed VP1 protein is indicated with an arrow. The PageRuler^TM^ Prestained Protein Ladder (Thermo Fischer Scientific) was included as a marker in lanes M in **b**, **c**. The N-terminal histidine tagged VP1 protein was detected with anti-histidine antibodies by western blot analysis among insoluble proteins that were separated under denaturing conditions and transferred to a PVDF membrane (**c**)

A 615-bp *VP1* gene of FMDV SAT2/ZIM/7/83 was amplified (Fig. 1a) and cloned in-frame into the pET28a vector for the expressed protein to contain an N-terminal histidine tag in transformed *E. coli* (BL21) DE3 cells. A 26-kDa product corresponding to the expected molecular mass of the expressed fusion protein was detected in the insoluble fraction after cell lysis (Fig. 1b) and reacted specifically with anti-histidine antibodies in a western blot (Fig. 1c).

### VP1 production in shake flasks

**Fig. 2:**
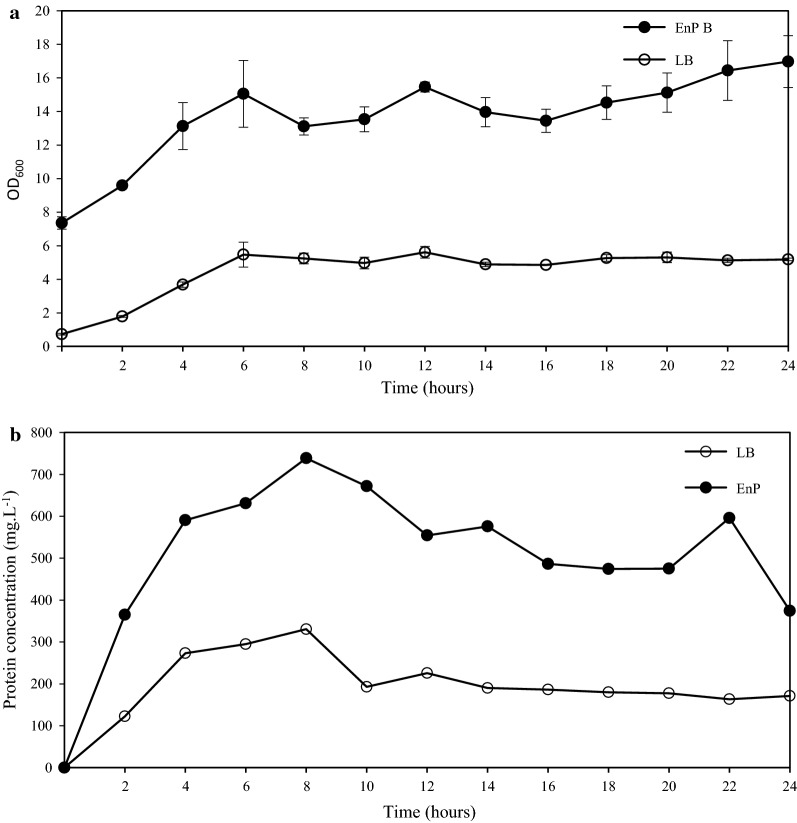
A graph showing the growth of EC-VP1 in shake flasks measured at OD_600_ (**a**) and VP1 protein expressed in EC-VP1 (**b**) in LB and EnPresso B media measured over 24 h post-induction. The OD_600_ was measured bi-hourly in triplicate (error bars indicate the standard deviation) and a pooled sample was analyzed for protein concentrations that were produced over 24 h

Production of VP1 was monitored bi-hourly in a time course study for 24 h post-induction in LB and EnPresso^®^ B media, respectively (Fig. 2). LB medium allows bacteria to grow in a batch mode, whereas the latter medium allows bacteria to grow in a fed-batch mode where the carbon source from the complex medium is released over time.

In LB medium, cultures were induced for protein production at an OD_600_ of 0.6 (time = 0 h). Thereafter, there was exponential growth of EC-VP1 to an OD_600_ of 6, followed by a stationary phase. This coincides with protein yields that increased and peaked at 330 mg L^−1^ at 8 h post-induction, followed by a decline and thereafter a maintenance of recombinant VP1 concentrations at ~ 190 mg L^−1^ until the end of the study.

In EnPresso B medium, cultures were induced for protein expression at an OD_600_ of 7.4. Recombinant VP1 concentrations were highest at 739 mg L^−1^ at 8 h post-induction (at an OD_600_ of 13), preceded by a higher OD_600_ of 15 at 6 h post-induction. In both LB and EnPresso^®^ B media, the VP1 protein was expressed as inclusion bodies. On average, the OD values of EC-VP1 grown in EnPresso^®^ B medium were at all points during protein production twice or more than EC-VP1 grown in LB. Within 2 h of induction, protein yields in LB and EnPresso^®^ B medium were at 122 mg L^−1^ and 365 mg L^−1^ respectively. In LB medium, yields peaked 8 h post-induction at 330 mg L^−1^, then decreased to 190 mg L^−1^ and remained at this concentration until the end of the study. In EnPresso^®^ B, yields also peaked 8 h post-induction at 739 mg L^−1^ (55% more than in LB) and by the end of the study the yields had decreased to 374 mg L^−1^.

Optimal conditions for expression of the recombinant VP1 was in EnPresso^®^ B medium harvested 8 h post-induction with 1 mM IPTG.

### VP1 production in fed-batch cultures

**Fig. 3:**
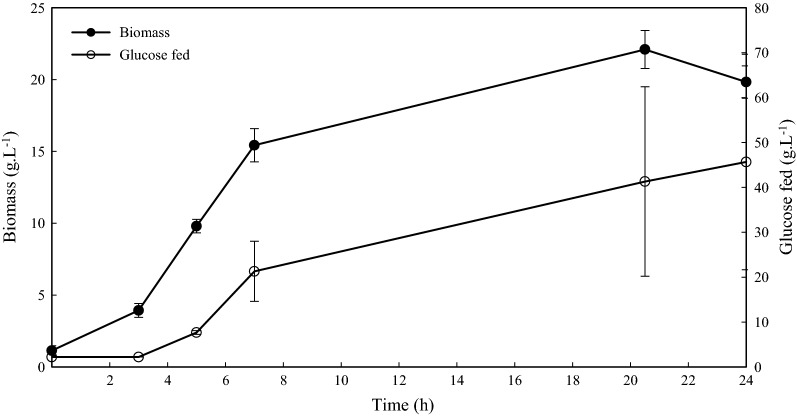
A graph showing growth of EC-VP1 and glucose fed during the production of VP1 in fed-batch fermentations. Infors fermenters (n = 3) with 1.5 L media were each inoculated with 100 mL of actively growing culture at an OD_600_ of 0.6. The cultures were induced for protein production with a final concentration of 1 mM IPTG at 7 h post-inoculation. Error bars represent the standard deviation

The EC-VP1 bacterial strain was cultured by fed-batch fermentation in 2 L bioreactors as described under “Materials and methods” section, and the results are presented in Fig. 3 and summarized in Table 1. The initial charge of glucose was consumed in 3 h, followed by a glucose feed. The EC-VP1 cultures were induced for protein production at 7 h post-inoculation with a final concentration of 1 mM IPTG. Pre-induction, the growth rate of the cultures was 0.432 h^−1^, with a DCW of 15.4 g L^−1^ and the OD_600_ was 40. During the induction phase the growth rate of the cultures reduced to 0.015 h^−1^, resulting in 2.15 g L^−1^ of product from a biomass of 19.8 g L^−1^ DCW (OD_600_ of 74) at harvest. The specific and volumetric productivities were 0.005 g g^−1^ h^−1^ and 0.090 g L^−1^ h^−1^, respectively. On average, 47.7 g L^−1^ of glucose was fed during the course of the fermentation.

**Table 1:** A summary of parameters that were measured in the production of recombinant VP1 by fed-batch fermentation

Dry cell weight (g L^−1^)		Final OD_600_ (600 nm)	Growth rate		Glucose fed (g L^−1^)	Protein titer (g L^−1^)	Productivity	
At induction	Final		Pre-induction	Post-induction			Specific (g g^−1^ h^−1^)	Volumetric (g L^−1^ h^−1^)
15.4	19.8	74	0.432	0.015	47.7	2.15	0.005	0.090

### Specificity

**Fig. 4:**
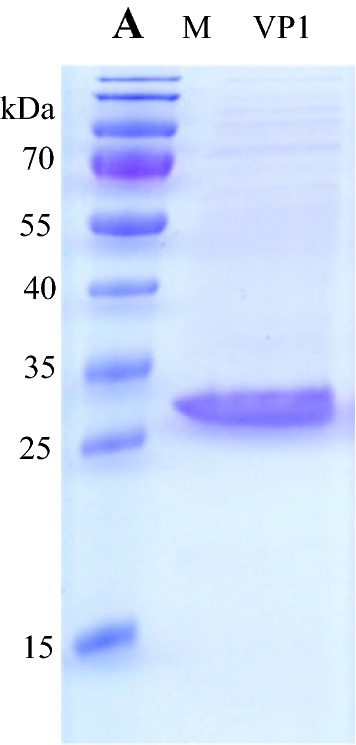
An image of a 15% polyacrylamide gel showing recombinant VP1 protein that was extracted, isolated and refolded, resulting in 73% pure protein. Loaded in lane M is the PageRuler^TM^ Prestained Protein Ladder (Thermo Fischer Scientific)

**Fig. 5:**
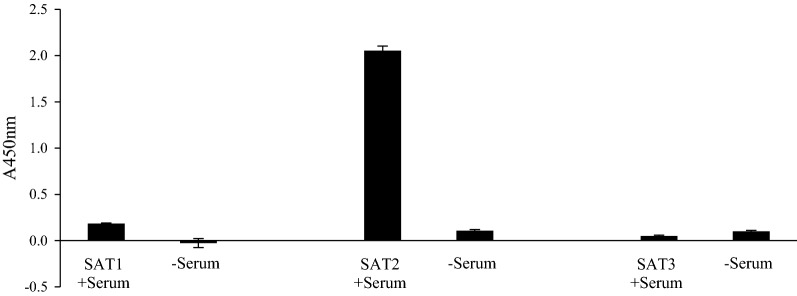
A graph illustrating the antigenicity and specificity of the recombinant VP1 as measured in a direct ELISA where the expressed VP1 protein was immobilized, then incubated in octuplicate with sera from guinea pigs that were immunized with FMDV SAT1, 2 and 3 viruses, respectively. Serum of naïve guinea pigs were included as controls (error bars represent the standard deviation)

Following extraction, isolation and refolding, recombinant VP1 protein was 73% pure (Fig. 4). The specificity of the VP1 was evaluated with sera from each of the SAT serotypes by ELISA. For this, naïve serum (−serum) and immune sera (+serum) of guinea pigs that were vaccinated with FMDV SAT1/SAR/9/81, SAT2/ZIM/7/83 and SAT3/KNP10/90 were reacted with immobilized recombinant VP1 in a direct ELISA, respectively. Chromogen-conjugated anti-guinea pig antibodies reacted with specificity resulting in a high signal with the SAT2 serum, and to a much lesser extent with the SAT1 and SAT3 sera (Fig. 5). This result confirms the antigenicity as well as the specificity of the recombinantly expressed SAT2 VP1 to serotype-specific antibodies.

## Discussion

Factors that are known to improve the solubility of proteins such as fusing the target protein to negatively charged fusion partners, co-expression of the target protein with chaperone sets, lower growth temperatures and inducer concentrations, using different media, expression of the protein in the *E. coli* pLysS strain were evaluated for VP1 expression in *E. coli*. These factors did not influence the solubility of the VP1 protein in this study (data not shown). Although the protein remained insoluble, the EnPresso^®^ B medium resulted in 45% more product at 8 h post-induction compared to LB broth (Fig. 2). Other researchers have also reported recombinant protein yield increases when using EnPresso^®^ B compared to LB broth (Ta et al. 2015; Zarschler et al. 2013). The ratio of protein yield per cell was, however, similar for EnPresso^®^ B medium and LB broth. This is similar to the observation by Zarschler et al. (2013), where the EnPresso^®^ medium outperformed LB broth in the expression of a single domain antibody 7C12, in *E. coli* BL21 (DE3) cells. In that study, protein titers of 13 mg L^−1^ were obtained from a culture of OD_600_ 1.4 grown in LB; compared to titers of 130 mg L^−1^ from OD_600_ 13.1. The added advantage of the EnPresso^®^ B medium is its ability to mimic fed-batch type production at small scale and provide insights into fed-batch fermentation performance. This is unlike LB, which resembles batch-like culture conditions.

In fed-batch fermentations VP1 protein yields were not followed during production, and only measured at harvest. There is a need to monitor recombinant protein production during the production process to understand when to harvest after induction to obtain maximum yield. As such, a comparative analysis of production in EnPresso^®^ B medium and fed-batch fermentations cannot be made in this article. Notwithstanding, in EnPresso^®^ B medium peak production of 739 mg L^−1^ at an OD_600_ of 13 was achieved. In comparison, a yield of 2.15 g L^−1^ at an OD_600_ of 74 in fed-batch fermentations was achieved. Protein production in EnPresso^®^ B medium was thus superior to fed-batch fermentation, and with simpler relative preparation steps.

During product formation, the growth rate of the EC-VP1 bacterial strain in fed-batch fermentation decreased from 0.432 to 0.015 (97% decrease). This is attributed to the metabolic load on the recombinant host as most energy and nutrients are diverted to heterologous protein production from cellular growth (Curless et al. 1990; Neubauer et al. 2003). At harvest, the protein yields were 2.15 g L^−1^ from 19.8 g L^−1^ DCW, which is comparable to yields of 3.8 g L^−1^ protein from 63 g L^−1^ of DCW obtained from the fusion of the VP1 epitope (aa 134–173) to GST at an OD_600_ of 156 in 5-L fermentors (Jung et al. 2013).

Recombinantly expressed VP1 proteins of several serotypes have been shown to be serotype specific. As such, they have been applied in the development of serotyping assays (Suryanarayana et al. 1999). From available literature, the use of serotype O VP1 protein for serotyping has been broadly studied, followed by Asia1. In this study, the specificity of a SAT2 VP1 protein was evaluated against SAT1, SAT2 and SAT3 viruses. The recombinantly expressed VP1 reacted with specificity with serum from a guinea pig that was vaccinated with FMDV SAT2, and negligibly to SAT1 and SAT3. The three SAT types co-occur in the Southern Africa and East Africa, so a diagnostic assay that can unequivocally distinguish them without using virus as reagent or in raising antibodies would be invaluable to the region. Challenges that would have to be overcome for such a test are the high antigenic diversity among SAT viruses. This is not an unsurmountable task as serotype A topotypes, considered to be among the most antigenically diverse in the world, could be detected with VP1 peptides in ELISA (Ko et al. 2010).

To the best of our knowledge this is one of a few reports on the expression of the entire unfused VP1 of the FMDV in *E. coli;* and the only one on the expression of a SAT2 VP1 protein in *E. coli* in shake flasks, upscaled to fermentation. Recombinant VP1 and peptides have been evaluated for the serotyping of the Euro-Asian serotypes, but not for the SAT serotypes. The protein was expressed as inclusion bodies that were isolated, denatured and refolded to react with high specificity to viral antigenic sites. This presents prospects for further evaluation of recombinantly expressed VP1 of SAT viruses to be used in serotyping and diagnostic assays.

## Supplementary information

**Additional file 1: Fig. S1.** Sequence alignment of the FMDV SAT2/ZIM/7/83 *VP1* gene with the *E. coli* codon optimized gene.

## Abbreviations

aa: amino acid
BSA: bovine serum albumin
DCW: dry cell weight
DO: dissolved oxygen
ELISA: enzyme-linked immunosorbent assay
FMD: foot-and-mouth disease
FMDV: foot-and-mouth disease virus
HRP: horseradish peroxidase
IPTG: isopropyl β-d-1-thiogalactopyranoside
LB: Luria–Bertani
OD: optical density
PAGE: polyacrylamide gel electrophoresis
SAT: Southern African Territories
SDS: sodium dodecyl sulfate
