# Supplementary material for: Production of foot-and-mouth disease virus SAT2 VP1 protein
Source: AMB Express. 2020 Jan 7;10:2. doi: 10.1186/s13568-019-0938-7 (PMC6946773; doi:10.1186/s13568-019-0938-7)
Supplement: Supplementary file 1 — Additional file 1: Fig. S1. Sequence alignment of the FMDV SAT2/ZIM/7/83 VP1 gene with the E. coli codon optimized gene. [file 13568_2019_938_MOESM1_ESM.docx]

*VP1*Gene GTCGTGACGACCGACCCTTCGACCCACGGTGGTGCTGTCACGGAGAAGAAACGTGTGCAC

*VP1*CodonOptimized GTTGTTACCACCGACCCGTCTACCCACGGTGGTGCGGTTACCGAAAAAAAACGTGTTCAC

** ** ** ******** ** ************** ** ** ** ** ******** ***

*VP1*Gene ACAGACGTGGCATTCGTCATGGACAGATTCACCCATGTTCTGACAAATAGAACCGCGTTC

*VP1*CodonOptimized ACCGACGTTGCGTTCGTTATGGACCGTTTCACCCACGTTCTGACCAACCGTACCGCGTTC

** ***** ** ***** ****** * ******** ******** ** * *********

*VP1*Gene GCGGTTGACTTGATGGACACCAACGAGAAGACCCTGGTAGGCGGCCTGCTGCGTGCGGCC

*VP1*CodonOptimized GCGGTTGACCTGATGGACACCAACGAAAAAACCCTGGTTGGTGGTCTGCTGCGTGCGGCG

********* **************** ** ******** ** ** **************

*VP1*Gene ACCTACTATTTCTGTGACCTGGAAATTGCCTGCCTTGGCGAACACGAACGCGTGTGGTGG

*VP1*CodonOptimized ACCTACTACTTCTGCGACCTGGAAATCGCGTGCCTGGGTGAACACGAACGTGTTTGGTGG

******** ***** *********** ** ***** ** *********** ** ******

*VP1*Gene CAGCCAAACGGGGCACCGCGGACAACCACGCTTCGCGACAACCCCATGGTGTTTTCACAC

*VP1*CodonOptimized CAGCCGAACGGTGCGCCGCGTACCACCACCCTGCGTGACAACCCGATGGTTTTCTCTCAC

***** ***** ** ***** ** ***** ** ** ******** ***** ** ** ***

*VP1*Gene AACAACGTCACGCGTTTTGCTGTCCCGTACACCGCGCCACACCGGCTGCTATCAACCAGA

*VP1*CodonOptimized AACAACGTTACCCGTTTCGCGGTTCCGTACACCGCGCCGCACCGTCTGCTGTCTACCCGT

******** ** ***** ** ** ************** ***** ***** ** *** *

*VP1*Gene TACAACGGTGAGTGCAAGTACACGCAGCAGTCCACTGCCATTCGCGGTGACCGTGCCGTC

*VP1*CodonOptimized TACAACGGTGAATGCAAATACACCCAGCAGTCTACCGCGATCCGTGGTGACCGTGCGGTT

*********** ***** ***** ******** ** ** ** ** *********** **

*VP1*Gene TTGGCCGCAAAGTACGCCAACACCAAACACAAACTCCCGTCTACCTTCAACTTCGGCCAC

*VP1*CodonOptimized CTGGCGGCGAAATACGCGAACACCAAACACAAACTGCCGTCTACCTTCAACTTCGGTCAC

**** ** ** ***** ***************** ******************** ***

*VP1*Gene GTGACCGCCGACAAACCAGTCGACGTTTACTACCGGATGAAGAGGGCGGCAGTCTACTGT

*VP1*CodonOptimized GTTACCGCGGACAAACCGGTTGACGTTTACTACCGTATGAAACGTGCGGCGGTTTACTGC

** ***** ******** ** ************** ***** * ***** ** *****

*VP1*Gene CCAAGACCTCTCCTCCCTGGCTACGACCACGCAGACAGGGACAGGTTTGACAGCCCCATT

*VP1*CodonOptimized CCGCGTCCGCTGCTGCCGGGTTACGACCACGCGGACCGTGACCGTTTCGACTCTCCGATC

** * ** ** ** ** ** *********** *** * *** * ** *** ** **

*VP1*Gene GGTGTTGAGAAACAA

*VP1*CodonOptimized GGTGTTGAAAAACAG

******** *****

Figure 1: Sequence alignment of the FMDV SAT2/ZIM/7/83 *VP1* gene with the *E. coli* codon optimized gene.
